# Nitric oxide from inflammatory origin impairs neural stem cell proliferation by inhibiting epidermal growth factor receptor signaling

**DOI:** 10.3389/fncel.2014.00343

**Published:** 2014-10-28

**Authors:** Bruno P. Carreira, Maria I. Morte, Ana I. Santos, Ana S. Lourenço, António F. Ambrósio, Caetana M. Carvalho, Inês M. Araújo

**Affiliations:** ^1^Centre for Neuroscience and Cell Biology, University of CoimbraCoimbra, Portugal; ^2^Regenerative Medicine Program, Department of Biomedical Sciences and Medicine, University of AlgarveFaro, Portugal; ^3^Centre for Molecular and Structural Biomedicine, CBME/IBB, University of AlgarveFaro, Portugal; ^4^Centre of Ophthalmology and Vision Sciences, Institute for Biomedical Imaging and Life Sciences (IBILI), Faculty of Medicine, University of CoimbraCoimbra, Portugal

**Keywords:** inflammation, microglia, nitric oxide, neural stem cells, cell proliferation

## Abstract

Neuroinflammation is characterized by activation of microglial cells, followed by production of nitric oxide (NO), which may have different outcomes on neurogenesis, favoring or inhibiting this process. In the present study, we investigated how the inflammatory mediator NO can affect proliferation of neural stem cells (NSCs), and explored possible mechanisms underlying this effect. We investigated which mechanisms are involved in the regulation of NSC proliferation following treatment with an inflammatory stimulus (lipopolysaccharide plus IFN-γ), using a culture system of subventricular zone (SVZ)-derived NSCs mixed with microglia cells obtained from wild-type mice (iNOS^+/+^) or from iNOS knockout mice (iNOS^-/-^). We found an impairment of NSC cell proliferation in iNOS^+/+^ mixed cultures, which was not observed in iNOS^-/-^ mixed cultures. Furthermore, the increased release of NO by activated iNOS^+/+^ microglial cells decreased the activation of the ERK/MAPK signaling pathway, which was concomitant with an enhanced nitration of the EGF receptor. Preventing nitrogen reactive species formation with MnTBAP, a scavenger of peroxynitrite (ONOO^-^), or using the ONOO^-^ degradation catalyst FeTMPyP, cell proliferation and ERK signaling were restored to basal levels in iNOS^+/+^ mixed cultures. Moreover, exposure to the NO donor NOC-18 (100 μM), for 48 h, inhibited SVZ-derived NSC proliferation. Regarding the antiproliferative effect of NO, we found that NOC-18 caused the impairment of signaling through the ERK/MAPK pathway, which may be related to increased nitration of the EGF receptor in NSC. Using MnTBAP nitration was prevented, maintaining ERK signaling, rescuing NSC proliferation. We show that NO from inflammatory origin leads to a decreased function of the EGF receptor, which compromised proliferation of NSC. We also demonstrated that NO-mediated nitration of the EGF receptor caused a decrease in its phosphorylation, thus preventing regular proliferation signaling through the ERK/MAPK pathway.

## INTRODUCTION

Brain inflammation was shown to be detrimental to neurogenesis (Ekdahl et al., 2003; Monje et al., 2003). However, the inflammatory factors underlying the negative effects of inflammation on the formation of new brain cells are not fully identified. NSCs and precursors proliferate throughout life in two regions of the adult central nervous system, namely in the SVZ of the lateral ventricles and the subgranular zone (SGZ) of the dentate gyrus of the hippocampus, as reviewed by Ming and Song (2011). Newborn cells can differentiate into various cell types, including neurons and glia (Eriksson et al., 1998; Gage, 2000; Alvarez-Buylla et al., 2002). The proliferation of NSC in the SVZ or SGZ, an initial step in the neurogenic process, increases following several insults to the brain, such as ischemic stroke (Arvidsson et al., 2002) or seizures (Parent et al., 1997), which might be part of the mechanisms supporting self-repair (Lowenstein and Parent, 1999; Curtis et al., 2007). Thus, the identification of potential therapeutic targets for the modulation of endogenous or exogenously induced neurogenesis is of great interest (Taupin, 2005; Madhusoodanan and Murad, 2007). Microglial cells are highly dynamic sensors that continually scan the surrounding tissue (Davalos et al., 2005; Nimmerjahn et al., 2005). Upon an injury, or change in the environment, microglia promptly respond with morphological and biochemical changes, producing and releasing a plethora of signaling molecules such as NO (Kettenmann et al., 2011).

Nitric oxide is a highly reactive diffusible signaling molecule, with pleiotropic effects in biological systems, having a half-life of few seconds. Upon neurotoxic, traumatic, and inflammatory damage in the mammalian brain, NO is formed in high amounts, following the expression of the iNOS (Chao et al., 1992; Galea et al., 1992; Nathan and Xie, 1994; Murphy, 2000; Calabrese et al., 2009). The increasing list of functions for NO from inflammatory origin includes bactericidal action, control of cerebral blood flow, regulation of endocytosis and vesicle recycling, and modulation of neurotransmission (Lane and Gross, 1999; Ignarro, 2000; Calabrese et al., 2007). One of the main mechanisms by which NO exerts its cellular functions is at the level of post-translational protein modification (Guix et al., 2005). Protein nitration results from the reaction of ONOO^-^, originated by reaction of NO with superoxide, with amino acidic residues such as tryptophan and tyrosine. Moreover, *S*-nitrosylation occurs when NO reacts with thiol groups of several amino acidic residues, particularly with cysteine. Both nitration and *S*-nitrosylation typically lead to alterations in protein function, with different outcomes in the cellular metabolism (Hanafy et al., 2001). In fact, reactive nitrogen and oxygen species are important factors in the inflammatory responses mediated by microglial cells (Rock et al., 2004). On the other hand, inflammation has different effects on neurogenesis and, particularly when inflammation occurs following tissue damage, it inhibits the neurogenic process (Ekdahl et al., 2003, 2009; Kempermann and Neumann, 2003; Monje et al., 2003).

Neural progenitor cells in the SVZ are anatomically in close proximity to NO-producing cells (Matarredona et al., 2005). Several studies have suggested that NO inhibits proliferation of NSCs under physiological conditions (Packer et al., 2003; Moreno-Lopez et al., 2004; Matarredona et al., 2005). In isolated NSC from the SVZ, supraphysiological concentrations of NO also inhibit NSC proliferation and promote differentiation of precursors into astrocytes (Covacu et al., 2006; Torroglosa et al., 2007; Bergsland et al., 2014). Torroglosa et al. (2007) have suggested that NO modulates the tyrosine kinase activity of EGF receptor (EGFR), although the precise underlying molecular mechanisms remain unclear. To date, the signaling pathways involved in the proliferative effect of NO following brain injury and the molecular mechanisms of its antiproliferative effect were not identified.

In the present study, we investigated how NO produced by microglia in inflammatory conditions can affect proliferation of NSCs, and assessed the mechanisms underlying the effects of NO on proliferation. To better understand whether and how NO mediates the detrimental effects of inflammation on NSC proliferation, we used a mixed culture system of SVZ-derived stem cells cultured with microglia isolated from wild-type mice (iNOS^+/+^) or knockout mice (iNOS^-/-^). We show, for the first time, that inflammatory NO produced by microglia has an antiproliferative effect through the decreased activation of the ERK/MAPK signaling pathway, an event for which the nitration of the EGFR plays an essential role, impairing NSC proliferation.

## MATERIALS AND METHODS

### MATERIALS

Dulbecco’s Modified Eagle’s Medium:F-12 nutrient mixture (D-MEM/F-12, with GlutaMAX^TM^-I), B27 supplement, trypsin-EDTA solution (0.05% trypsin, 1 mM EDTA in HBSS), gentamicin, antibiotics (10,000 units/ml of penicillin, 10 mg/ml streptomycin), and trypsin (1:250) were purchased from Life Technologies (Carlsbad, CA, USA). Deoxyribonuclease 1 (DNase-1), BrdU, phenylmethylsufonyl fluoride, dithiothreitol, orthovanadate, chymostatin, leupeptin, antiparin, pepstatin A, trypan blue, LPS, and alkaline phosphatase-linked anti-rabbit secondary antibody were purchased from Sigma Chemical (St Louis, MO, USA). BCATM Protein Assay kit was obtained from Pierce (Rockford, IL, USA). EGF, bFGF, Click-iT^®^ EdU Alexa Fluor^®^ 647 HCS Assay, Hoechst 33342, anti-mouse IgG conjugated with Alexa Fluor 594 or 488, and anti-rabbit IgG conjugated with Alexa Fluor 633, 594, or 488 secondary antibodies were purchased from Life Technologies (Carlsbad, CA, USA). Cellulose acetate (0.45 μm) was obtained from Corning Inc. (Lowell, MA, USA). M-CSF and IFN-γ were purchased from Peprotech (London, UK) and NOC-18 from Alexis Biochemicals (San Diego, CA, USA). BSA and MnTBAP were obtained from Calbiochem (San Diego, CA, USA). The antibody against 3-NT conjugated with agarose beads and FeTMPyP were purchased from Cayman Chemical (Tallinn, Estonia). Rabbit anti-GFAP and DAKO fluorescent mounting medium were obtained from DakoCytomation (Glostrup, Denmark). Rat anti-mouse BrdU was obtained from Oxford Biotechnology and rat anti-mouse CD11b from Serotec (Oxford, UK). Mouse anti-nestin, rabbit anti-iNOS and mouse anti-GAPDH were purchased from BD Transduction (San Jose, CA, USA). Mouse anti-3-nitrotyrosine was purchased from Upstate Biotechnology (Charlottesville, VA, USA), while rabbit anti-EGFR, rabbit anti-phospho-Tyr1173-EGFR, rabbit anti-phospho-ERK1/2 and mouse anti-ERK1/2 were purchased from Cell Signaling (Danvers, MA, USA). Rabbit anti-nestin was obtained from Santa Cruz Biotechnology (Santa Cruz, CA, USA) and mouse anti-Sox-2 from R&D Systems (Minneapolis, MN, USA). Griess Reagent System was obtained from Promega (Madison, WI, USA). Polyvinylidene difluoride (PVDF) membranes, enhanced chemifluorescence (ECF) reagent and alkaline phosphatase-linked anti-rabbit and anti-mouse secondary antibodies were obtained from Amersham Pharmacia Biotech (Buckinghamshire, UK). Other reagents used in immunoblotting experiments were purchased from BioRad (Hercules, CA, USA).

### ANIMALS

C57BL/6J (iNOS^+/+^) mice or B6.129P2-*Nos2^tm1Lau^*/J (iNOS^-/-^) were obtained from Charles River (Barcelona, Spain) or The Jackson Laboratory (Bar Harbor, ME, USA), respectively, and kept with food and water *ad libitum* in a 12 h dark:light cycle. All experiments were performed in accordance with NIH and European guidelines (86/609/EEC) for the care and use of laboratory animals. In addition, all the people working with animals have received appropriate education (FELASA course) as required by the Portuguese authorities. Furthermore, the animals were housed in our licensed animal facility (International Animal Welfare Assurance number 520.000.000.2006). This study is part of a grant approved and financed by the Foundation for Science and Technology, (FCT, Portugal), that approved the animal experimentation described (reference PTDC/SAU-NEU/102612/2008).

### PRIMARY MICROGLIAL CELL CULTURES

Primary glial cultures were prepared from the brains of 0 to 3-day-old C57BL/6J (iNOS^+/+^) or B6.129P2-*Nos2^tm1Lau^*/J (iNOS^-/-^) mice according to the method of Giulian and Baker (1986). Briefly, the brains were removed from the skull, following decapitation, and placed in dissection medium composed of Ca^2+^- and Mg^2+^-free HBSS (137 mM NaCl, 5.36 mM KCl, 0.44 mM KH_2_PO_4_, 0.34 mM Na_2_PO_4_ . 2H_2_O, 4.16 mM NaHCO_3_, 5 mM glucose, 1 mM sodium pyruvate, 10 mM HEPES, pH 7.4), supplemented with 0.25% gentamicin. The enveloping meninges and the cerebellum were discarded and the cortex tissue was mechanically dissociated and digested with trypsin (0.1%) and DNase 1 (0.001%) in Ca^2+^- and Mg^2+^- free HBSS for 20 min, at 37°C. Cells were seeded in 75 cm^2^ flasks coated with poly-L-lysine, at a density of 0.2 × 10^6^ cells/cm^2^ and cultured in D-MEM/F-12 with GlutaMAX^TM^-I supplemented with 10% FBS, 0.25% gentamicin and 0.25 ng/ml M-CSF, at 37°C and 95% air-5% CO_2_ in a humidified incubator. Culture medium was changed every 3–4 days and confluency was achieved after 10–14 DIV. Microglia were detached from the mixed glial cultures 3–10 days after reaching confluency, by shaking at 200 rpm for 2 h, and collected from the supernatant by centrifuging at 1500 rpm, for 5 min. Cells were then seeded for 3 days at a density of 0.035 × 10^6^ cells/cm^2^ onto 16-mm diameter glass coverslips, for immunocytochemistry assays, or on 12-well plates, for preparation of lysates, both coated with poly-L-lysine, in serum-free medium, without M-CSF. Next, cultures were treated with an acute inflammatory stimulus (except the controls): 100 ng/ml LPS plus 0.5 ng/ml IFN-γ, for 24 h (Saura et al., 2003).

### SUBVENTRICULAR ZONE CELL CULTURES

Neural stem cell cultures were obtained from the SVZ of postnatal day 0–3 wild-type or transgenic C57BL/6J mice expressing enhanced green fluorescent protein (GFP) under the control of the actin promoter, as previously described by Carreira et al. (2010). The SVZ-derived NSCs were allowed to develop as primary neurospheres in a 95% air-5% CO_2_ humidified atmosphere at 37°C, during 7 days. Next, neurospheres were collected, dissociated and plated for 5 days on 16-mm diameter glass coverslips, for immunocytochemistry assays, or on 12-well plates, coated with poly-L-lysine, in the same medium as above, without growth factors, for preparation of lysates.

### MIXED CELL CULTURES

Green fluorescent protein-positive SVZ neurospheres were collected, dissociated and plated together with iNOS^+/+^ or iNOS^-/-^ microglial cell cultures (from now on denominated as iNOS^+/+^ or iNOS^-/-^ mixed cultures, respectively) on 16-mm diameter glass coverslips, for immunocytochemistry assays, or on 12-well plates, for preparation of lysates, both coated with poly-L-lysine, and kept in fresh D-MEM/F-12 with GlutaMAX^TM^-I medium, supplemented with 1% B27, 0.25% gentamicin, 10 ng/ml EGF and 10 ng/ml bFGF, at 37°C and 95% air-5% CO_2_ in a humidified incubator, for 3 days. GFP-positive SVZ neurospheres were also dissociated and seeded alone, for 3 days, on 16-mm diameter coverslips or on 12-well plates, coated with poly-L-lysine, and cultured in the same medium as above, for control experiments. Cultures were treated with 100 ng/ml LPS plus 0.5 ng/ml IFN-γ, for 24 h. Control cultures were left untreated. The cell-permeable superoxide dismutase mimetic and ONOO^-^ scavenger MnTBAP (100 μM; Szabo et al., 1996) or the ONOO^-^ decomposition catalyst FeTMPyP (50 μM; Misko et al., 1998), when used, were added 30 min before LPS plus IFN-γ and kept throughout the incubation period.

### EXPERIMENTAL TREATMENTS IN SVZ-DERIVED NEURAL STEM CELL CULTURES

Subventricular zone-derived NSC were exposed to the NO donor NOC-18 (100 μM) for 48 h. MnTBAP (100 μM) and FeTMPyP (50 μM), were added 30 min before NOC-18 and kept throughout the incubation period.

### DETECTION OF BrdU INCORPORATION IN SVZ-DERIVED NEURAL STEM CELL CULTURES

To analyze proliferation of SVZ-derived NSCs, 10 μM BrdU was added to the cultures 16 h prior to fixation (Alvaro et al., 2008; Carreira et al., 2010). Nuclei that incorporated BrdU in this time-window were detected by immunofluorescence, as detailed next. Following 20 min fixation with 4% paraformaldehyde/4% sucrose in PBS (0.1 M), the cells were permeabilized with 1% Triton X-100 for 5 min, and DNA was denaturated by treatment with 1 M HCl for 30 min, at 37°C. Non-specific binding was blocked with 3% BSA in 0.2% Tween-20 in PBS (PBS-T) for 1 h, and then BrdU-positive cells were labeled with a rat anti-BrdU antibody (1:50) for 90 min, at room temperature. The cells were then incubated with a secondary antibody goat anti-rat IgG conjugated with Alexa Fluor 594 (1:200), for 1 h, at room temperature. Nuclei were stained with Hoechst 33342 (1 μg/ml) for 5 min. Coverslips were mounted on glass slides using DAKO fluorescence mounting medium. The images were acquired in a laser-scanning microscope LSM 510 META (Zeiss, Jena, Germany) or in a fluorescence microscope (Axioskop 2 Plus, Zeiss, Jena, Germany). The number of BrdU-positive nuclei was counted in 8–10 randomly selected fields for each coverslip (in a total of ∼900–1,200 cells per coverslip), and the data were expressed as percentage of the total number of live cells. A minimum of three independent experiments, from NSC cultures prepared from different animals, was analyzed for each condition.

### DETECTION OF EdU INCORPORATION

Neural stem cell proliferation was also assessed by incorporation of the thymidine analog EdU, which is incorporated into DNA of dividing cells during S phase (Buck et al., 2008; Cappella et al., 2008; Chehrehasa et al., 2009). EdU (10 μM) was added to the cultures 4 h prior to fixation. Nuclei that incorporated EdU in this time-window were detected by immunofluorescence, as follows. Following 20 min fixation with 4% paraformaldehyde/4% sucrose in PBS (0.1 M), the cells were washed with 3% BSA/PBS and then permeabilized with 0.5% Triton X-100/PBS for 15 min, at room temperature. The cells were then incubated with the Click-iT reaction cocktail [1x at 87.5% (v/v) Click-iT Reaction Buffer, 2% (v/v) CuSO_4_, 0.05% (v/v) fluorescent azide (Alexa Fluor 647), and 1x at 10% (v/v) Reaction Buffer Additive], protected from light. Cells were then washed twice in 3% BSA/PBS and an immunocytochemistry was performed as detailed next.

The number of EdU-positive nuclei was counted in 8–10 randomly selected fields for each coverslip (in a total of ∼900–1,200 cells per coverslip), and the data were expressed as percentage of the total number of live cells. A minimum of three independent experiments was analyzed for each condition.

### IMMUNOCYTOCHEMISTRY

Following fixation and permeabilization, non-specific binding was blocked with 3% BSA. Cells were incubated with the primary antibodies for 90 min, at room temperature. After rinsing with PBS, the cells were incubated with the appropriate secondary antibodies for 1 h (1:200, anti-mouse, anti-rabbit or anti-rat IgGs conjugated with Alexa Fluor 488, 594, or 633), at room temperature. All antibodies were prepared in blocking solution. Nuclei were labeled with Hoechst 33342 (1 μg/ml) for 5 min, after incubation with the secondary antibodies. Coverslips were mounted on glass slides, the cells were visualized using a fluorescence microscope (Axioskop 2 Plus, Zeiss, Jena, Germany) and the images were acquired with the Axiovision software (release 4.7) or in a laser scanning fluorescence microscope LSM 510 META (Zeiss, Jena, Germany). The primary antibodies and the concentrations used were: mouse anti-Sox-2, 1:100; rabbit anti-nestin, 1:100; rabbit anti-GFAP, 1:400; mouse anti-nestin, 1:500; rat anti-mouse-CD11b, 1:200; rabbit anti-iNOS, 1:200.

### EVALUATION OF NITRIC OXIDE PRODUCTION

Nitric oxide production was indirectly assessed by measuring the concentration of nitrites in the culture medium (Green et al., 1982), in primary microglia cultures, SVZ-derived NSC cultures or mixed cell cultures. A commercial kit from Promega was used, and the standard protocol provided by the supplier was followed. The concentration of nitrite for each sample was calculated from a standard curve using a sodium nitrite solution and data were expressed in μM.

### WESTERN BLOT ANALYSIS

Cells were lysed in 50 mM Tris-HCl, 10 mM EGTA, 1% Triton X-100 and 2 mM MgCl_2_, supplemented with 100 μM phenylmethylsufonyl fluoride, 1 mM dithiothreitol, 1 μg/ml chymostatin, 1 μg/ml leupeptin, 1 μg/ml antiparin, 5 μg/ml pepstatin A, 1 mM sodium orthovanadate, 50 mM NaF, pH 7.4, at 4°C. Protein concentration was determined by the bicinchoninic acid (BCA) method, and the samples were used for Western blot analysis, after adding 6x concentrated sample buffer [0.5 M Tris, 30% glycerol, 10% sodium dodecyl sulfate (SDS), 0.6 M dithiothreitol, 0.012% bromophenol blue] and heating, for 5 min, at 95°C. Equal amounts of protein were separated by electrophoresis on SDS-polyacrylamide gels, and transferred electrophoretically to PVDF membranes. These were then blocked for 1 h at room temperature, in Tris-buffered saline (137 mM NaCl, 20 mM Tris-HCl, pH 7.6) containing 0.1% Tween-20 (TBS-T) and 3% BSA. Incubations with primary antibodies (anti-iNOS or anti-GAPDH, 1:500; rabbit anti-phospho-Tyr1173-EGFR, rabbit anti-EGFR, rabbit anti-phospho-ERK1/2 and mouse anti-ERK1/2, 1:1,000) in TBS-T with 1% BSA were performed overnight, at 4°C. Next, the membranes were incubated for 1 h at room temperature with alkaline phosphatase-linked secondary antibodies (anti-rabbit or anti-mouse IgG, 1:20,000) in TBS-T with 1% BSA. After extensive washing in TBS-T with 0.5% BSA, immunoreactive bands were visualized in the VersaDoc 3000 imaging system (BioRad, Hercules, CA, USA), following incubation of the membrane with ECF reagent for 5 min.

### IMMUNOPRECIPITATION

Following the various experimental treatments, as detailed in figure legends, the cultures were lysed in 20 mM Tris-HCl, 100 mM NaCl, 2 mM EDTA, 2 mM EGTA, supplemented with 100 μM PMSF, 1 mM dithiothreitol, 1 μg/ml chymostatin, 1 μg/ml leupeptin, 1 μg/ml antiparin, 5 μg/ml pepstatin A, 1 mM sodium orthovanadate, 50 mM NaF, pH 7.0, at 4°C. Protein concentration was determined by the BCA method, and the samples were used for immunoprecipitation of nitrated proteins, using an antibody against 3-NT conjugated with agarose beads. Briefly, equal amounts of sample (250 μg of protein) were incubated with the antibody overnight at 4°C, and then with the beads for 2 h at room temperature. Following rinsing, the supernatant was discarded and the beads were suspended in 2x concentrated sample buffer, boiled for 5 min, and centrifuged using Spin-X centrifuge tube filters (0.45 μm cellulose acetate), to separate the beads from the immunoprecipitates. Equal volumes of immunoprecipitate were loaded onto SDS-PAGE gels, and Western blotted as described above against the EGF receptor.

### STATISTICAL ANALYSIS

Data are expressed as mean ± SEM. Statistical significance was determined by using two-tailed *t*-tests, one-factor or two-factor analysis of variance (ANOVA) as appropriate, followed by *post hoc* Bonferroni’s or Dunnet’s tests, as indicated in the figure legends and in the text. Differences were considered significant when *p* < 0.05.

## RESULTS

### INFLAMMATORY STIMULATION INCREASES iNOS EXPRESSION AND NO FORMATION

Neural stem cells were isolated from the SVZ and cultured as floating aggregates, also referred as neurospheres. Neurospheres were then dissociated and plated on poly-L-lysine-coated coverslips for 3–5 days, and characterized at this stage. Staining against the transcription factor Sox-2, and against nestin, a neural precursor cell marker, was performed. The percentage of double-labeled cells was approximately 70%, which suggests that the majority of cells remained undifferentiated after plating as shown previously by our group (Carreira et al., 2010).

**FIGURE 1:**
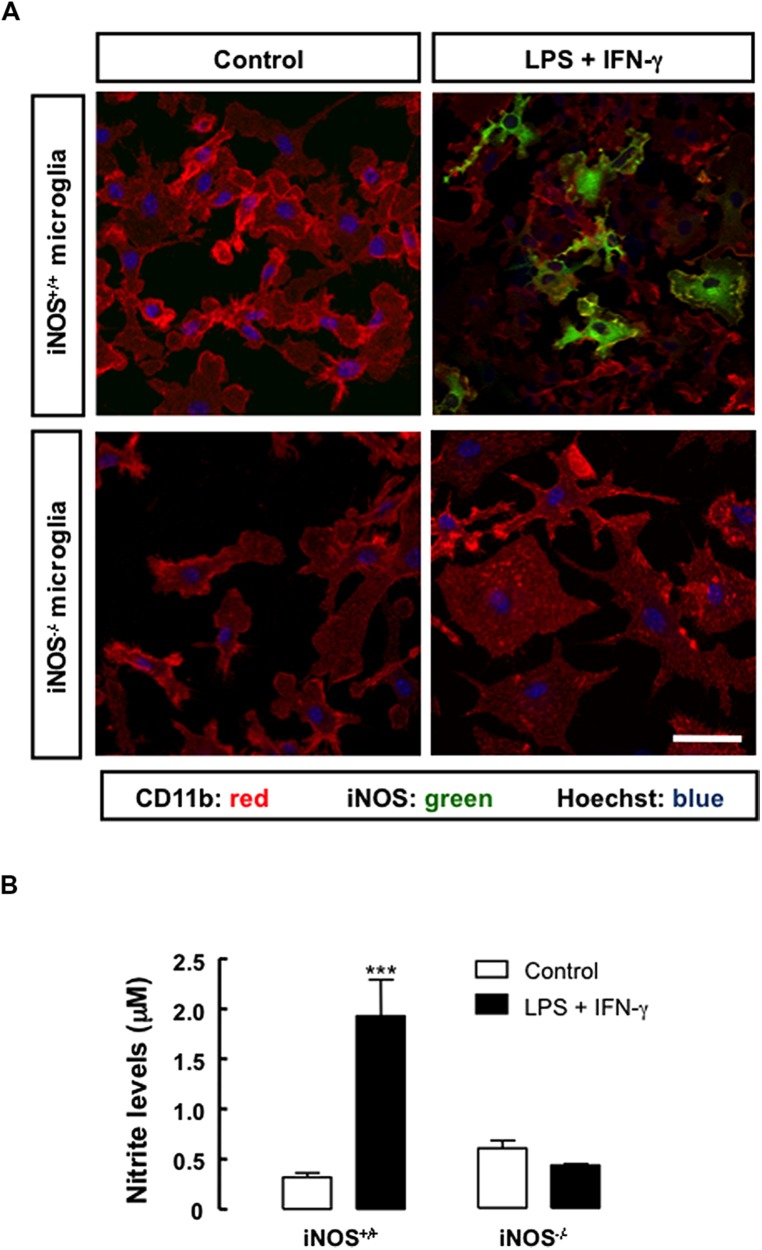
**Nitric oxide production and iNOS expression in primary microglial cell cultures under inflammatory conditions. (A)** Exposure to LPS plus IFN-γ for 24 h triggered the expression of iNOS in iNOS^+/+^ microglia (CD11b-positive cells), but not in iNOS^-/-^ microglia. Nuclei were labeled with Hoechst 33342. Scale bar: 20 μm. **(B)** Production of NO, which was indirectly measured by quantification of nitrite levels in the culture media, following treatment with LPS plus IFN-γ for 24 h, in primary iNOS^+/+^ or iNOS^-/-^ microglial cell cultures. Two-way ANOVA; ***p < 0.001, significantly different from control.

Microglial cells were seeded on poly-L-lysine-coated coverslips for 3 days and challenged with an inflammatory stimulus, LPS (100 ng/ml) plus IFN-γ (0.5 ng/ml), for 24 h. Cultures were then characterized by evaluating microglial cells morphology, iNOS expression and NO production. In iNOS^+/+^ microglial cell cultures, exposure to LPS plus IFN-γ increased iNOS immunoreactivity, but not in iNOS^-/-^ microglial cell cultures (**Figure 1A**), as expected. Concomitantly, following treatment with LPS plus IFN-γ, both iNOS^+/+^ and iNOS^-/-^ microglial cells exhibited an activated morphology with ovaloid cytoplasm, marked cellular hypertrophy and retraction of processes. In order to estimate the amount of NO produced by activated microglia in culture, we assessed NO production by measuring nitrite levels in the culture media following challenging with LPS plus IFN-γ. Nitrite levels were higher in treated iNOS^+/+^ microglial cell cultures (1.95 ± 0.3 μM, *p* < 0.001), than in untreated cultures (0.32 ± 0.1 μM), corresponding to a 6-fold increase in NO production above control levels. In iNOS^-/-^ microglial cell cultures, treatment with LPS plus IFN-γ did not significantly change NO levels, as compared to untreated cultures (**Figure 1B**).

**FIGURE 2:**
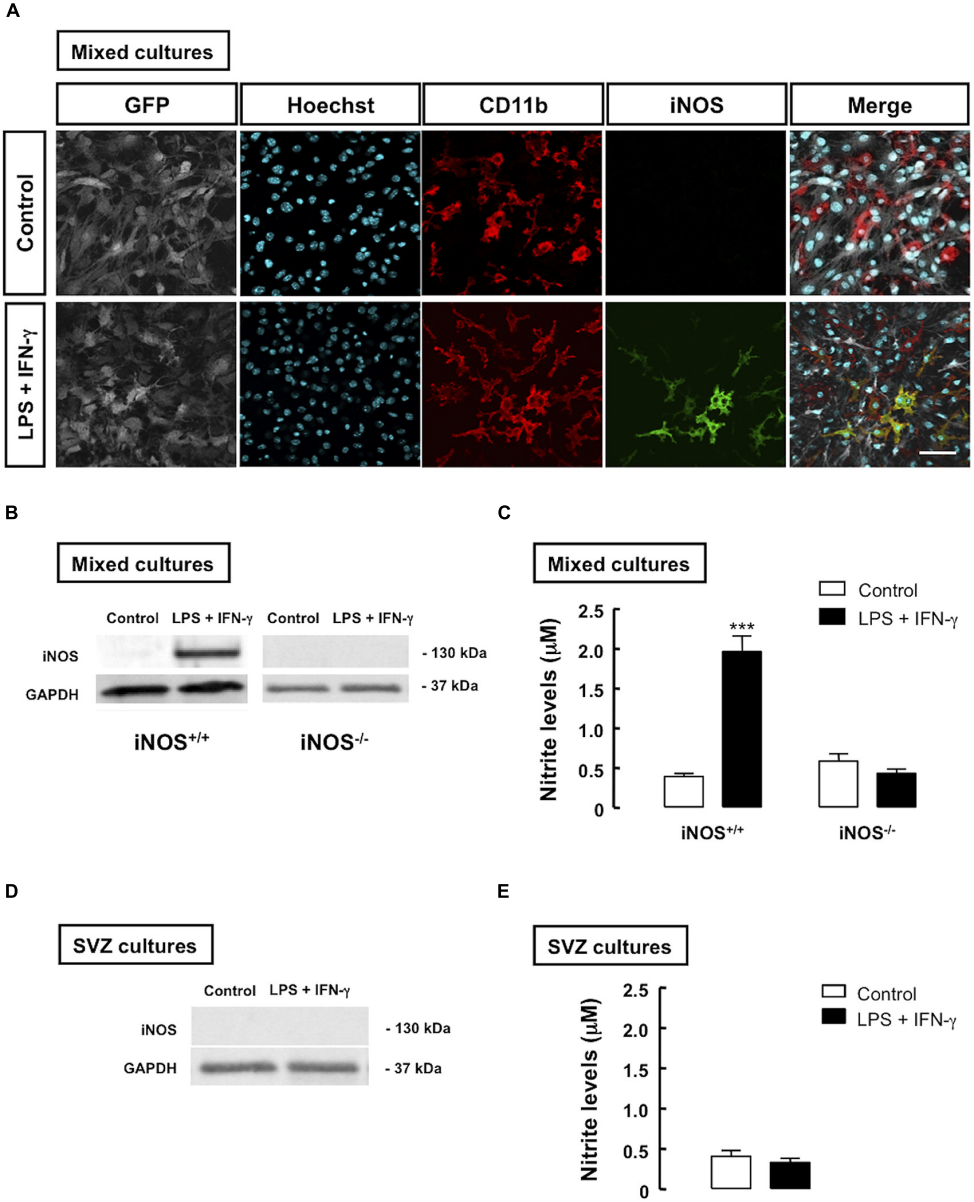
**Inflammatory stimulation increases iNOS expression and NO production in microglia co-cultured with SVZ cells. (A)** Presence of iNOS-positive cells (green) in mixed cultures of iNOS^+/+^ microglia (CD11b-positive cells) together with GFP-positive SVZ cells, following treatment with LPS plus IFN-γ, for 24 h, as compared to controls (upper panels). Nuclei were labeled with Hoechst 33342 (blue). Scale bar: 20 μm. **(B)** Detection of iNOS in mixed cultures of SVZ cells with iNOS^+/+^ microglia, but not with iNOS^-/-^ microglia, after treatment with LPS plus IFN-γ for 24 h, as assessed by Western blotting. GAPDH was used as a loading control. **(C)** Production of NO, following 24 h treatment with LPS plus IFN-γ, in mixed cultures. Two-way ANOVA; ***p < 0.001, significantly different from control. **(D)** Absence of iNOS immunoreactivity in SVZ cells cultured alone, upon exposure to LPS plus IFN-γ for 24 h. GAPDH was used as loading control. **(E)** NO production after 24 h of treatment with LPS plus IFN-γ, in SVZ-derived NSC cultures. Two-tailed *t*-test; *p* > 0.05.

To investigate how inflammation, particularly NO formed due to microglial activation, could affect the proliferation of SVZ-derived NSCs, mixed cultures were prepared as described in Section “Materials and Methods.” We evaluated the expression of iNOS in iNOS^+/+^ mixed cultures, following treatment with LPS plus IFN-γ by immunocytochemistry (**Figure 2A**) and Western blot analysis (**Figure 2B**). In iNOS^-/-^ mixed cultures, iNOS was not detected, as assessed by Western blotting, even after the inflammatory stimulus (**Figure 2B**). Furthermore, when measuring nitrite levels in the culture media, an increase in NO production was observed in iNOS^+/+^ mixed cultures (1.96 ± 0.2 μM, *p* < 0.001) following treatment with LPS plus IFN-γ, as compared to control cultures (0.39 ± 0.1 μM). On the contrary, treatment with LPS plus IFN-γ for 24 h did not significantly change NO levels in iNOS^-/-^ mixed cultures, as compared to untreated cultures (**Figure 2C**).

To determine whether SVZ-derived NSCs contributed to the increase in iNOS levels observed in mixed cell cultures, we also evaluated the presence of iNOS in SVZ-derived NSC alone, and observed a complete absence of immunoreactivity against iNOS following treatment with LPS plus IFN-γ in these cultures (**Figure 2D**). Moreover, treatment with LPS plus IFN-γ for 24 h did not significantly change NO levels in SVZ-derived NSC cultures, as compared to untreated cultures, as assessed by evaluating nitrite levels in cultures (**Figure 2E**).

### NO FROM INFLAMMATORY ORIGIN EXERTS AN ANTIPROLIFERATIVE EFFECT ON SVZ-DERIVED NEURAL STEM CELLS

**FIGURE 3:**
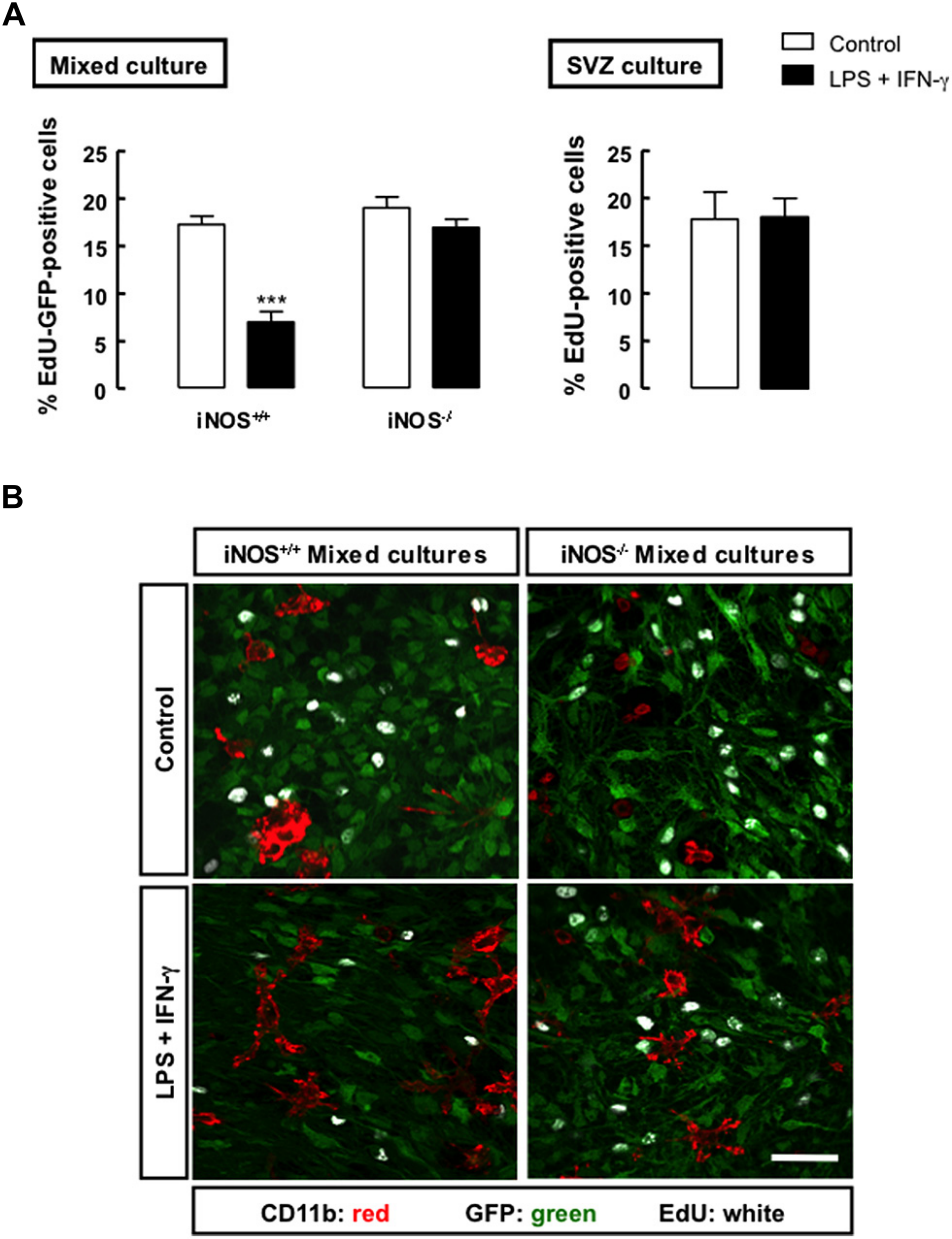
**Nitric oxide from microglial origin impairs the proliferation of SVZ cells. (A)** Incorporation of EdU in mixed or SVZ-derived NSC cultures following treatment with LPS plus IFN-γ for 24 h. Two-way ANOVA (mixed culture); ***p < 0.001, significantly different from control. Two-tailed *t*-test (SVZ culture); *p* > 0.05. **(B)** Representative images of the incorporation of EdU (white) in GFP-positive SVZ cells (green) cultured together with iNOS^+/+^ or iNOS^-/-^ microglia (CD11b-positive cells; red), following 24 h exposure to LPS plus IFN-γ. Scale bar: 20 μm.

To determine how NO released from microglial cells affected the proliferation of SVZ-derived NSCs, we evaluated the incorporation of EdU in iNOS^+/+^ or iNOS^-/-^ mixed cultures, following treatment with LPS plus IFN-γ. In iNOS^+/+^ mixed cultures, we observed that EdU incorporation significantly decreased to 7.0 ± 1.09% (*p* < 0.001), 24 h following exposure to LPS plus IFN-γ, as compared to control cultures (17.3 ± 0.88%), but the same stimulus had no effect in EdU incorporation either in iNOS^-/-^ mixed cultures or in SVZ-derived NSC cultures alone (**Figure 3A**).

Moreover, we observed that SVZ-derived NSCs, but not microglia, incorporated EdU, as illustrated by the presence of immunoreactivity against EdU in the GFP-positive SVZ cells, but not in microglial cells, strongly suggesting that these are the dividing cells in mixed cultures (**Figure 3B**). We also confirmed that LPS plus IFN-γ for 24 h did not affect cell viability in iNOS^+/+^ and in iNOS^-/-^ mixed cultures, or in SVZ-derived NSC cultures (**Tables 1** and **2**, respectively).

**Table 1:** Cell viability evaluation in iNOS^+/+^ and iNOS^–/–^ mixed cultures following exposure to LPS plus IFN-γ for 24 h.

	Treatment	% GFP-live cells	% GFP-dead cells
iNOS^+/+^	Control LPS plus IFN-γ LPS plus IFN-γ plus MnTBAP LPS plus IFN-γ plus FeTMPyP	87.5 ± 1.4 90.3 ± 0.8 (n.s.) 90.7 ± 1.5 (n.s.) 88.8 ± 1.9 (n.s.)	12.5 ± 0.6 9.7 ± 1.1 (n.s.) 9.3 ± 0.8 (n.s.) 11.2 ± 0.9 (n.s.)
iNOS^-/-^	Control LPS plus IFN-γ LPS plus IFN-γ plus MnTBAP LPS plus IFN-γ plus FeTMPyP	92.9 ± 1.5 91.5 ± 1.4 (n.s.) 89.8 ± 1.7 (n.s.) 88.7 ± 1.6 (n.s.)	7.1 ± 1.1 8.5 ± 0.9 (n.s.) 10.2 ± 1.0 (n.s.) 11.3 ± 0.8 (n.s.)

	**Treatment**	**% CD11b-live cells**	**% CD11b-dead cells**

iNOS^+/+^	Control LPS plus IFN-γ LPS plus IFN-γ plus MnTBAP LPS plus IFN-γ plus FeTMPyP	99.2 ± 0.3 99.5 ± 0.3 (n.s.) 98.7 ± 0.4 (n.s.) 99.0 ± 0.7 (n.s.)	0.8 ± 0.3 0.5 ± 0.1 (n.s.) 1.3 ± 0.5 (n.s.) 1.0 ± 0.6 (n.s.)
iNOS^-/-^	Control LPS plus IFN-γ LPS plus IFN-γ plus MnTBAP LPS plus IFN-γ plus FeTMPyP	98.7 ± 0.4 99.0 ± 0.2 (n.s.) 98.9 ± 0.5 (n.s.) 99.3 ± 0.7 (n.s.)	1.3 ± 0.1 1.0 ± 0.3 (n.s.) 1.1 ± 0.4 (n.s.) 0.7 ± 0.4 (n.s.)

*Cell viability was assessed by analysis of nuclear morphology. Cells were considered dead when nuclei were condensed/fragmented and brightly stained with Hoechst 33342. Cells presenting regular nuclear morphology and light nuclear stain with bright nucleoli were considered live cells. n.s. (non-significant) *p* > 0.05, not different from the control, one-way ANOVA (Dunnett’s post-test).*

**Table 2:** Cell viability evaluation in SVZ-derived NSC cultures.

	Treatment	% live cells	% dead cells
24 h	Control LPS plus IFN-γ	92.7 ± 0.9 90.5 ± 0.5 (n.s.)	7.3 ± 0.5 9.5 ± 0.9 (n.s.)
48 h	Control NOC-18 NOC-18 plus MnTBAP MnTBAP	80.0 ± 2.0 77.5 ± 2.8 (n.s.) 80.2 ± 1.7 (n.s.) 82.6 ± 1.3 (n.s.)	20.0 ± 1.0 22.5 ± 1.2 (n.s.) 19.8 ± 0.9 (n.s.) 17.4 ± 0.8 (n.s.)

*Cell viability was assessed by analysis of nuclear morphology. Cells were considered dead when nuclei were condensed/fragmented and brightly stained with Hoechst 33342. Cells presenting regular nuclear morphology and light nuclear stain with bright nucleoli were considered live cells. n.s. (non-significant) p > 0.05, not different from the control, one-way ANOVA (Dunnett’s post-test).*

### NO FROM INFLAMMATORY ORIGIN DECREASES THE ACTIVATION OF THE ERK/MAPK SIGNALING PATHWAY

**FIGURE 4:**
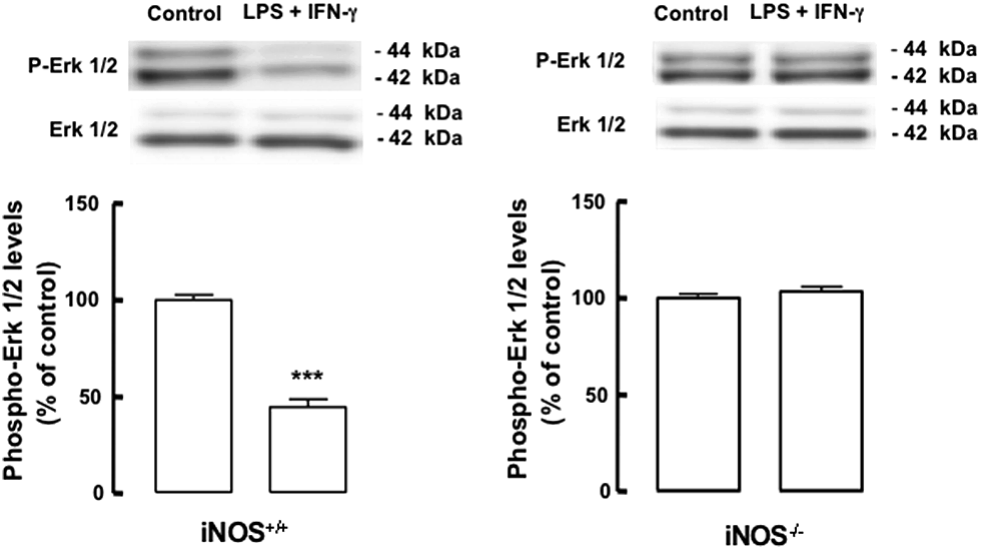
**Nitric oxide from microglia origin decreases the activation of the ERK1/2 signaling pathway.** Decreased phosphorylation of ERK1/2 in iNOS^+/+^ mixed culture, but not in iNOS^-/-^ mixed culture lysates, following exposure to LPS plus IFN-γ. One-way ANOVA (Bonferroni’s post-test). ***p < 0.001, significantly different from control.

We next investigated the possible mechanism underlying the observed antiproliferative effect of inflammation in mixed cultures. The main regulatory pathway for NSC proliferation is the signaling cascade of MAPK, particularly the ERK1/2 pathway. Within this scenario, we evaluated whether inflammation, particularly NO from inflammatory origin, could affect the activation and signaling through the ERK/MAPK pathway. We found that LPS plus IFN-γ decreased the phosphorylation of ERK1/2 in iNOS^+/+^ mixed cultures (**Figure 4**, *p* < 0.001), whereas in iNOS^-/-^ mixed cultures no changes were observed in the levels of phospho-ERK1/2 following exposure to LPS plus IFN-γ (**Figure 4**).

**FIGURE 5:**
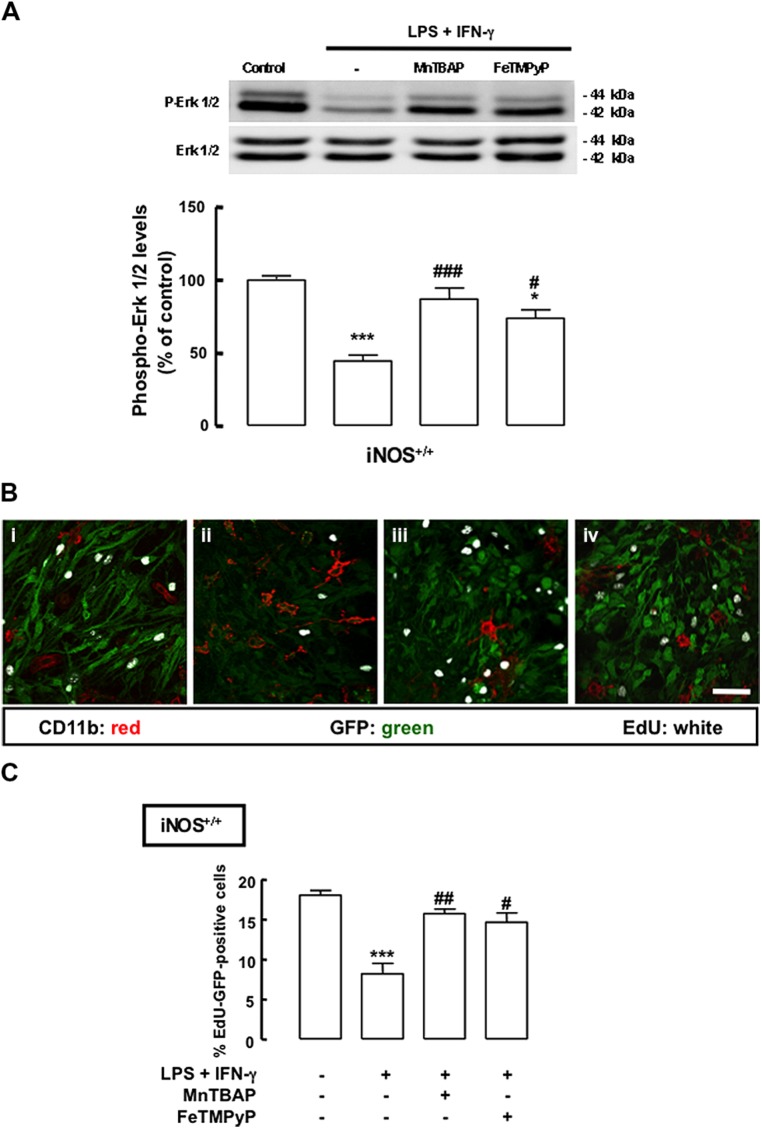
**Mn(III)tetrakis(4-benzoic acid)porphyrin chloride or FeTMPyP prevents the antiproliferative effect of NO. (A)** Decreased phosphorylation of ERK1/2 in iNOS^+/+^ mixed cultures, following exposure to LPS plus IFN-γ. Either MnTBAP or FeTMPyP partially prevented the decrease of ERK1/2 phosphorylation. One-way ANOVA (Bonferroni’s post-test). *p < 0.05 or ***p < 0.001, significantly different from control. ^###^*p* < 0.001 or ^#^*p* < 0.05, significantly different from LPS plus IFN-γ. **(B)** Representative images of the incorporation of EdU in SVZ cells cultured together with iNOS^+/+^ microglia, following 24 h exposure to LPS plus IFN-γ with or without MnTBAP (100 μM) or FeTMPyP (50 μM). (i) Control; (ii) LPS plus IFN-γ; (iii) LPS plus IFN-γ plus MnTBAP; (iv) LPS plus IFN-γ plus FeTMPyP. Scale bar: 20 μm. **(C)** Incorporation of EdU in iNOS^+/+^ mixed cultures upon treatment with LPS plus IFN-γ for 24 h, with or without MnTBAP (100 μM) or FeTMPyP (50 μM). One-way ANOVA (Bonferroni’s post-test); ***p < 0.001, significantly different from control; ^#^*p* < 0.05 or ^##^*p* < 0.01, significantly different from LPS plus IFN-γ.

To better understand whether the antiproliferative effect of inflammation in mixed cultures could be due to the production of highly reactive species, such as ONOO^-^, we used MnTBAP, a superoxide scavenger, thus preventing the production of ONOO^-^, and FeTMPyP, which catalyzes the degradation of ONOO^-^. We observed that the decreased phosphorylation of ERK1/2 in iNOS^+/+^ mixed cultures following treatment with LPS plus IFN-γ was prevented either by treatment with MnTBAP or FeTMPyP (**Figure 5A**, *p* < 0.001 or *p* < 0.05, respectively). Moreover, when MnTBAP or FeTMPyP were present during the inflammatory stimulus, cell proliferation was rescued to 15.8 ± 0.4% or 14.7 ± 0.8%, respectively, in iNOS^+/+^ mixed cultures (**Figures 5B,C**).

**FIGURE 6:**
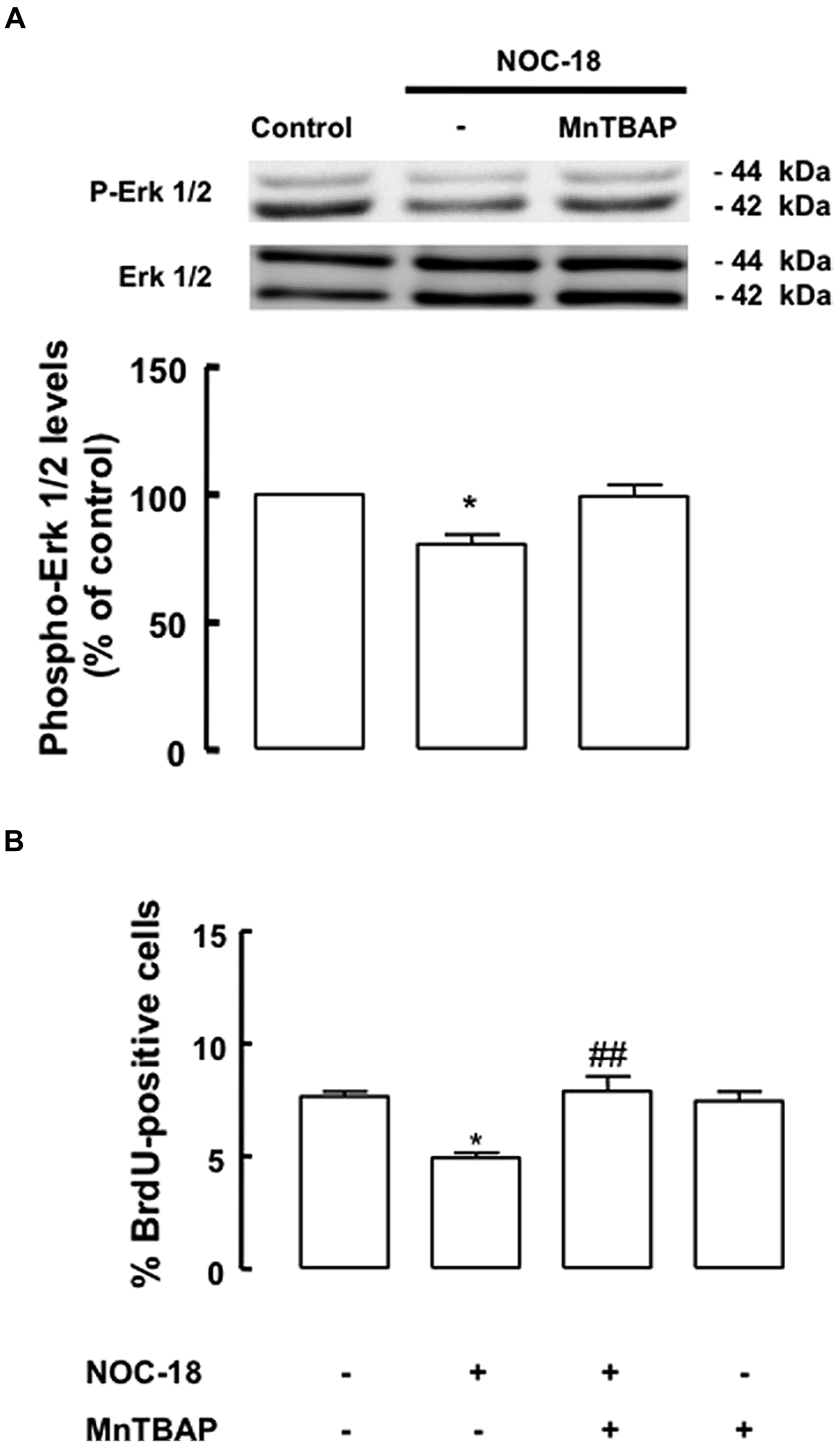
**High levels of NO decrease the activation of the ERK1/2 signaling pathway. (A)** High levels of NOC-18 (100 μM) decreased the phosphorylation of ERK1/2 in SVZ cell lysates. MnTBAP prevented the decrease of ERK1/2 phosphorylation. One-way ANOVA (Bonferroni’s post-test). *p < 0.05, significantly different from control. **(B)** MnTBAP (100 μM) prevented the antiproliferative effect of NOC-18 (100 μM) in SVZ cell cultures, as determined by BrdU incorporation. One-way ANOVA (Bonferroni’s post-test). *p < 0.05, significantly different from control. ^##^*p* < 0.01, significantly different from NOC-18.

We also investigated the effect of high levels of NO, an event occurring during inflammation, in the proliferation of SVZ-derived NSC cultures. For that, SVZ-derived NSCs were exposed to a NO donor, NOC-18 (100 μM), for 48 h. We observed a decreased phosphorylation of ERK1/2, following exposure to 100 μM NOC-18 for 48 h, which was prevented by MnTBAP (**Figure 6A**, *p* < 0.05). When assessing NSC proliferation by evaluating BrdU incorporation following treatment with NOC-18 (100 μM) for 48 h, we observed that NOC-18 significantly decreased BrdU incorporation to 4.9 ± 0.2% (*p* < 0.001), as compared to BrdU incorporation in control cultures (7.7 ± 0.2%). Interestingly, MnTBAP rescued cell proliferation (7.9 ± 0.66%), when present during the treatment with NOC-18 for 48 h (**Figure 6B**). Furthermore, we also confirmed that NOC-18 for 48 h did not affect cell viability in SVZ-derived NSC cultures (**Table 2**).

### INFLAMMATION INDUCES NITRATION OF THE EGF RECEPTOR AND DECREASES ITS PHOSPHORYLATION STATUS

**FIGURE 7:**
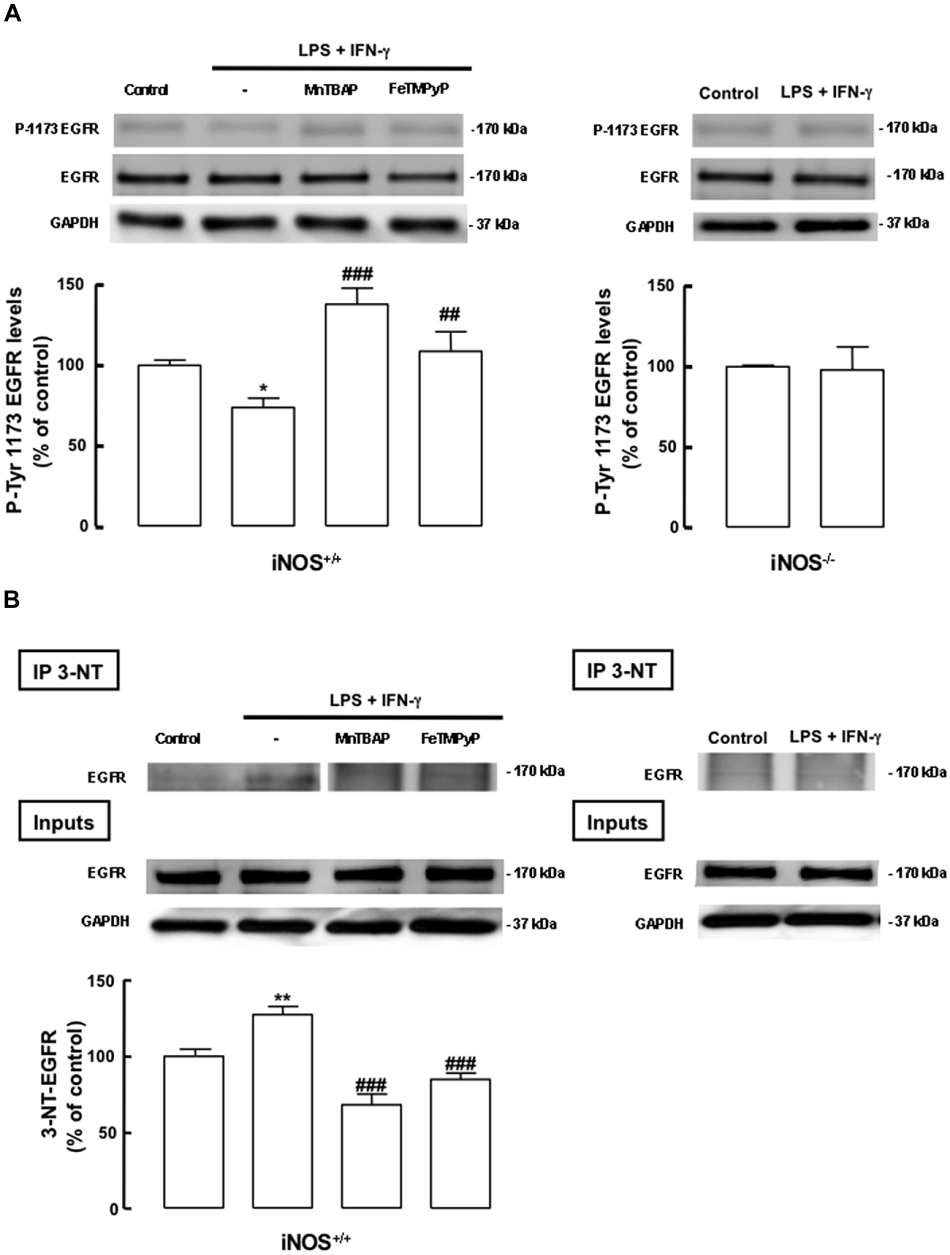
**Nitric oxide from inflammatory origin has an antiproliferative effect, via nitration and decreased phosphorylation of the EGF receptor, in iNOS^+/+^ mixed cultures. (A)** Decreased phosphorylation of tyrosine residues (P-Tyr 1173) in iNOS^+/+^ mixed culture, but not in iNOS^-/-^ mixed culture lysates, following exposure to LPS plus IFN-γ. Both MnTBAP or FeTMPyP partially prevented the decrease of tyrosine phosphorylation in the EGFR. EGFR or GADPH were used as loading controls. One-way ANOVA (Bonferroni’s post-test). *p < 0.05, significantly different from Control. ^###^*p* < 0.001 or ^##^*p* < 0.01, significantly different from LPS plus IFN-γ. Two-tailed *t*-test (iNOS^-/-^ mixed culture); *p* > 0.05. **(B)** Nitration of the EGFR in SVZ cells in mixed cultures with iNOS^+/+^ microglia, but not with iNOS^-/-^ microglia, following stimulation with LPS plus IFN-γ for 24 h. MnTBAP (100 μM) and FeTMPyP (50 μM) prevented the nitration of the EGFR in iNOS^+/+^ mixed cultures, following exposure to LPS plus IFN-γ for 24 h. Nitration was assessed by immunoblotting against the EGFR, after immunoprecipitation of the nitrated proteins (IP-3NT). EGFR or GAPDH were used as loading controls. One-way ANOVA (Bonferroni’s post-test). **p < 0.01, significantly different from Control. ^###^*p* < 0.001, significantly different from LPS plus IFN-γ.

We next evaluated whether the inflammatory stimulus induced the nitration of the EGF receptor in mixed cultures, an event that could be linked to the inhibition of the downstream signaling through the ERK/MAPK pathway in mixed cultures treated with LPS plus IFN-γ. Nitration of tyrosine residues of the EGFR was evaluated in iNOS^+/+^ and iNOS^-/-^ mixed cultures. In iNOS^+/+^ mixed cultures, treatment with LPS plus IFN-γ for 24 h increased the nitration of the EGFR in immunoprecipitates of nitrated proteins (127.4 ± 5.4%, *p* < 0.01), as compared to untreated cultures. Moreover, MnTBAP and FeTMPyP were able to prevent the nitration of the EGFR in these cultures, after incubation with LPS plus IFN-γ, reducing EGFR nitration to 68.2 ± 7.2% (*p* < 0.001) or 84.9 ± 2.4% (*p* < 0.001) of the control, respectively (**Figure 7A**, left pannel). On other hand, this effect was not observed in iNOS^-/-^ mixed cultures, where treatment with LPS plus IFN-γ did not cause increased nitration of the EGFR (**Figure 7A**, right panel).

Next, we analyzed the phosphorylation of the tyrosine residue 1173, which is involved in the activation of the EGFR. LPS plus IFN-γ caused a decrease in the phosphorylation status of the EGFR in iNOS^+/+^ mixed cultures, an event that was prevented either by MnTBAP or FeTMPyP (**Figure 7B**, left panel). As expected, in iNOS^-/-^ mixed cultures no changes were observed in the phosphorylation status of the EGFR following exposure to LPS plus IFN-γ for 24 h (**Figure 7B**, right panel).

## DISCUSSION

In this work, we demonstrated that inflammation has an antiproliferative effect on NSC. Furthermore, we show that the inhibitory effect of inflammation on the proliferation of NSC is mediated by NO, due to the decreased signaling through the ERK/MAPK pathway, subsequent to the nitration of the EGF receptor and decrease of its phosphorylation status.

The effect of inflammation on the proliferation of SVZ-derived NSCs was studied in this work by establishing mixed cultures of iNOS^+/+^ or iNOS^-/-^ microglial cells and SVZ-derived NSCs. These mixed cultures, when challenged with LPS plus IFN-γ, are a good system to mimic events occurring *in vivo* during an inflammatory response (Cacci et al., 2008) in the neurogenic niche, particularly NO production, thus allowing us to dissect the involved of NO and underlying mechanism in the antiproliferative effect of neuroinflammation. To study more directly the effect of NO in SVZ-derived NSCs, primary SVZ-derived cell cultures were exposed to high levels of the NO donor (NOC-18), an experimental condition that mimics pathophysiological conditions, particularly neuroinflammatory conditions, when high concentrations of NO can be found locally in the brain. For this purpose, SVZ-derived NSCs cultures were incubated with 100 μM NOC-18 for 48 h, since it is a long-acting NO donor with a half-life of ∼22 h (Keefer et al., 1996). At this concentration (100 μM), NOC-18 released NO mimicking what might be found in neuroinflammatory conditions. We did not observe any cytotoxic effect of NO in all the conditions tested in this study (Boje and Arora, 1992; Dawson et al., 1993, 1994; Bal-Price and Brown, 2001). In addition, we assessed the activation of the ERK/MAPK signaling pathway, which is involved in the regulation of SVZ-derived NSC proliferation downstream of EGFR (Reynolds and Weiss, 1996; Doetsch et al., 2002; Carreira et al., 2010, 2012). High levels of NO, achieved following treatment with NOC-18, decreased the phosphorylation of ERK1/2 in SVZ-derived NSC cultures, suggesting an inhibition of the signaling pathway controlling NSC proliferation after exposure to NO. MnTBAP prevented the decrease in the phosphorylation of ERK1/2 and was also able to rescue cell proliferation to control levels, suggesting that the antiproliferative effect of NO is mostly due to the formation of nitrogen reactive species, which compromises signaling through the MAPK pathway.

Furthermore, we show that LPS plus IFN-γ decreased the activation of the ERK pathway downstream of the EGFR in iNOS^+/+^ mixed cultures, which was prevented by MnTBAP and FeTMPyP. This event was not observed in iNOS^-/-^ mixed cultures, where no differences were found in the phosphorylation status of ERK following treatment with LPS plus IFN-γ. Here we hypothesized that the antiproliferative effect of inflammation is mediated by NO production, causing a decreased signaling through the ERK/MAPK pathway. This event might be mediated by intermediate formation of nitrogen reactive species, such as ONOO^-^, since high levels of NO can inhibit mitochondrial respiration and elicit superoxide production (Beltran et al., 2002; Moncada and Bolanos, 2006; Calabrese et al., 2009). Different mechanisms by which microglia impairs neurogenesis in inflammatory conditions have been reported, which involves the release of proinflammatory mediators, such as IL-1 (Ben-Hur et al., 2003), IL-6 (Ekdahl et al., 2003), IFN-γ (Butovsky et al., 2006), and TNF-alpha (Iosif et al., 2006; for review on this topic; see Sierra et al., 2014). In addition, in several pathophysiological conditions associated with inflammatory processes, activated inflammatory cells generate large amounts of reactive oxygen species such as superoxide, hydrogen peroxide and the hydroxyl radical, concomitantly with increased iNOS expression and production of large amounts of NO. NO and superoxide readily react to form ONOO^-^, which is an extremely reactive molecule (Beckman and Crow, 1993; Ischiropoulos and al-Mehdi, 1995; Pryor and Squadrito, 1995; Radi et al., 2001), also making it very difficult to experimentally measure the production of superoxide in conditions where NO is present. Nitration is an irreversible chemical modification occurring in proteins that results from the reaction of ONOO^-^ with amino acid residues, particularly tyrosine (Hanafy et al., 2001). Tyrosine nitration seriously affects tyrosine phosphorylation/dephosphorylation signaling cascades (Monteiro et al., 2008).

Indeed, in this work we also observed that exposure to a high concentration of NO caused the nitration of the EGF receptor in iNOS^+/+^ mixed cultures. We observed that the increased production of NO by iNOS^+/+^ microglia triggered by treatment with LPS plus IFN-γ is concomitant with increased nitration of the EGF receptor, which correlates with a decreased signaling through the ERK/MAPK pathway, leading to an antiproliferative effect on SVZ-derived NSCs. Moreover, lack of NO (by knockout of iNOS), prevented the nitration of the EGF receptor under inflammatory conditions. Indeed, in iNOS^-/-^ mixed cultures, although there was activation of microglial cells following treatment with LPS plus IFN-γ, which was characterized by the presence of morphologic changes, no differences in cell proliferation were found when compared to untreated cultures. Several studies have shown that the pharmacological inhibition of inflammation triggered by injection of LPS (Monje et al., 2003), or experimentally induced seizures (Ekdahl et al., 2003), can restore hippocampal neurogenesis. However, some pro-inflammatory mediators, such as interleukin-6 (IL-6), released by activated microglia, seem to be important contributors to the inhibition of SGZ neurogenesis (Vallieres et al., 2002; Monje et al., 2003). On the other hand, microglia can also release trophic factors (Batchelor et al., 1999), like brain-derived neurotrophic factor (BDNF), that has been reported to promote neurogenesis (Benraiss et al., 2001). Thus, activated microglia may have a beneficial or detrimental role depending on the stimulus, and local and temporal environmental changes during a lesion to the nervous system (Kettenmann et al., 2011).

It should be noted that in mixed cultures cells are in close contact, therefore, it is very likely that in iNOS^+/+^ mixed cultures, NO levels achieved locally can be even higher, as evidenced by the increased nitration of the EGF receptor following acute stimulation of microglial cells with LPS plus IFN-γ. Moreover, we showed that scavenging nitrogen reactive species formation, by MnTBAP or FeTMPyP treatment (Misko et al., 1998; Salvemini et al., 1998), restored basal cell proliferation and nitration of the EGF receptor was prevented in iNOS^+/+^ mixed cultures.

Our data further indicates that concomitantly with increased nitration in tyrosine residues of the EGF receptor, NO decreased the phosphorylation status of this receptor, possibly by inhibiting the tyrosine kinase activity of EGFR, which has over 15 tyrosine residues that can be phosphorylated and contribute to its activation. We demonstrate that nitration of tyrosine residues prevents the phosphorylation of such tyrosine residues in the EGF receptor, which is paramount for its activation and downstream signaling. In addition, MnTBAP or FeTMPyP prevented the decrease in the phosphorylation status of the EGF receptor in iNOS^+/+^ mixed cultures challenged with LPS plus IFN-γ. Altogether, these evidences support our hypothesis that the antiproliferative effect of inflammation is mediated by NO production. NO from inflammatory origin is involved in the decreased activation of the ERK/MAPK pathway, which is mainly caused by the nitration of EGFR receptor, which compromises its phosphorylation and further signaling. Some studies have reported that NO can have an antiproliferative effect by affecting the phosphorylation of the EGF receptor in fibroblasts (Estrada et al., 1997), neuroblastoma cells (Murillo-Carretero et al., 2002, 2009), and NSCs (Torroglosa et al., 2007). In fact, in neuroblastoma cells, it was shown that NO inhibits the EGF receptor by *S*-nitrosylation (Murillo-Carretero et al., 2009), but the inhibitory effect of NO produced by microglia (via inducible NO synthase) in NSC proliferation was only now addressed in our study.

To our knowledge, this is the first study showing nitration of the EGF receptor as the main cause to the impairment of the signaling through the ERK/MAPK pathway, particularly under inflammatory conditions, which compromise proliferation of NSCs. In Caco-2 cells (cell line obtained from human colon carcinoma), nitration of EGFR was first observed following the exposure to ONOO^-^ (Uc et al., 2003). Several other players of the EGFR signaling cascade are also likely to be susceptible to nitration, since they also have tyrosine residues that participate in EGFR signaling by phosphorylation/dephosphorylation and may, thus, be nitrated and inactivated. Although we did not address these other players in this work, it cannot be excluded that other participants in proliferation signaling, other than the EGF receptor and downstream signaling cascades, can be affected by NO-mediated nitration, particularly in inflammatory conditions. Moreover, the antiproliferative effect of acute inflammatory conditions may be related to the enhancement of cell differentiation, where NO is likely to be a player. There is evidence showing that NO from inflammatory origin is involved in astrogliogenesis (Covacu et al., 2006; Bergsland et al., 2014), neurogenesis in the SVZ (Walton et al., 2006), or oligodendrogenesis (Kempermann and Neumann, 2003; Butovsky et al., 2006).

In previous works by our group we reported that low levels of NO, although in the pathophysiological range, have a proliferative effect in SVZ-derived NSCs by targeting two pathways in a biphasic manner, initially mediated by the ERK/MAP kinase pathway (Carreira et al., 2010) and at later stages by the cGMP/PKG pathway (Carreira et al., 2012). In this work, we observed that higher levels of NO have an opposite effect by preventing regular proliferation signaling through the ERK/MAPK pathway through the nitration of the EGF receptor which caused a decrease in its phosphorylation. Although we did not test the effect of low dose of NO or contribution of cGMP/PKG in our mixed culture model, we cannot exclude a possible effect of high levels of NO in preventing regular signaling through this pathway, as already described by others in a pathological context both *in vitro* and *in vivo* (Zhang et al., 2002, 2012; for review on this topic; see Zhang et al., 2013). One of the mechanisms underlying NO toxicity in neurogenic niches includes ONOO^-^ formation, an event that has been described *in vivo* after ischemic stroke (Keynes and Garthwaite, 2004).

Overall, our findings highlight that control of the nitrergic system may be an important target in cell transplantation techniques, particularly in the case of grafting of NSC in lesioned areas. Published evidence demonstrates that surviving grafts are influenced by the innate immune response, particularly due to acute microglia activation, which significantly impacts the survival and fate of both endogenous and transplanted neural progenitor cells (Monje et al., 2002, 2003; Ekdahl et al., 2003; Ormerod et al., 2008). In this context, this work highlights a mechanism that may be involved in the deleterious effect of inflammation in the viability of grafted NSCs, which is mediated by high levels of NO produced by microglia via iNOS.

Nevertheless, we show that it is possible to prevent the antiproliferative effects of microglia during an acute inflammatory response, by preventing the formation of nitrogen reactive species, with could be used as a strategy to improve the efficiency of transplantation techniques. Overall, our work sheds new light into the mechanisms underlying the effects of NO on the proliferation of NSCs, and may help in steering research efforts toward understanding how modulation of the nitrergic system can be used to regulate proliferation of stem cells in a regenerative context.

## AUTHOR CONTRIBUTIONS

Maria I. Morte, Ana I. Santos, and Ana S. Lourenço contributed to conception and design of the work, acquisition, analysis and interpretation of data for the work, and drafting the manuscript. António F. Ambrósio has been involved in conception, analysis and interpretation of data, being also involved in drafting and revising the manuscript critically. Bruno P. Carreira, Caetana M. Carvalho, and Inês M. Araújo have been involved in the conception, design of the work, acquisition, analysis and interpretation of data, being also involved in drafting and revising the manuscript critically. All authors agree that all the questions related to the accuracy or integrity of the work have been appropriately investigated and resolved, giving final approval of the version to be published.

## Conflict of Interest Statement

The authors declare that the research was conducted in the absence of any commercial or financial relationships that could be construed as a potential conflict of interest.

## Abbreviations

bFGF: basic fibroblast growth factor
BSA: bovine serum albumin
BrdU: 5-bromo-2′-deoxyuridine
NOC-18: DETA-NONOate
DIV: days *in vitro*
EdU: 5-ethynyl-2′-deoxyuridine
EGF: epidermal growth factor
EGFR: epidermal growth factor receptor
ERK: extracellular signal-regulated kinase
FeTMPyP: 5,10,15,20-tetrakis(N-methyl-4′-pyridyl)porphinate iron (III) chloride
HBSS: Hank’s balanced salt solution
iNOS: inducible nitric oxide synthase
IFN-γ: interferon-gamma
LPS: lipopolysaccharide
MAPK: mitogen-activated protein kinases
M-CSF: macrophage colony stimulating factor
MnTBAP: Mn(III)tetrakis(4-benzoic acid)porphyrin chloride
NOC-18: (Z)-1-[N-(2-aminoethyl)-N-(2-ammonioethyl)amino]diazen-1-ium-1,2-diolate
NO: nitric oxide
3-NT: 3-nitrotyrosine
NSCs: neural stem cells
ONOO^-^: peroxynitrite
PBS: phosphate-buffered saline
SVZ: subventricular zone.
